# Lynch Syndrome: An Updated Review

**DOI:** 10.3390/genes5030497

**Published:** 2014-06-27

**Authors:** Rishabh Sehgal, Kieran Sheahan, Patrick R. O’Connell, Ann M. Hanly, Sean T. Martin, Desmond C. Winter

**Affiliations:** Centre for Colorectal Disease, St. Vincent’s University Hospital, Elm Park, Dublin 4, Ireland; E-Mails: rishsehgal@gmail.com (R.S.); K.Sheahan@st-vincents.ie (K.S.); Ronan.OConnell@ucd.ie (R.O.C.); annhanly@rcsi.ie (A.M.H.); drseanmartin@gmail.com (S.T.M.)

**Keywords:** Lynch syndrome, genetics, screening, history, CRC clinic

## Abstract

Lynch syndrome is one of the most common cancer susceptibility syndromes. Individuals with Lynch syndrome have a 50%–70% lifetime risk of colorectal cancer, 40%–60% risk of endometrial cancer, and increased risks of several other malignancies. It is caused by germline mutations in the DNA mismatch repair genes *MLH1*, *MSH2*, *MSH6* or *PMS2.* In a subset of patients, Lynch syndrome is caused by 3' end deletions of the *EPCAM* gene, which can lead to epigenetic silencing of the closely linked *MSH2*. Relying solely on age and family history based criteria inaccurately identifies eligibility for Lynch syndrome screening or testing in 25%–70% of cases. There has been a steady increase in Lynch syndrome tumor screening programs since 2000 and institutions are rapidly adopting a universal screening approach to identify the patients that would benefit from genetic counseling and germline testing. These include microsatellite instability testing and/or immunohistochemical testing to identify tumor mismatch repair deficiencies. However, universal screening is not standard across institutions. Furthermore, variation exists regarding the optimum method for tracking and disclosing results. In this review, we summarize traditional screening criteria for Lynch syndrome, and discuss universal screening methods. International guidelines are necessary to standardize Lynch syndrome high-risk clinics.

## 1. Introduction

Approximately 2%–5% of all colorectal cancers (CRC) arise from a defined inherited cancer syndrome [1]. Lynch syndrome (formerly known as hereditary nonpolyposis colorectal cancer, HNPCC) and familial adenomatous polyposis (FAP) are autosomal dominant genetic disorders that comprise the majority of these. Other less common familial CRC syndromes include the hamartomatous syndromes, hyperplastic polyposis syndromes, the autosomal recessive MYH-associated polyposis syndrome, and familial juvenile polyposis. Although the global incidence of heritable CRC is relatively low when compared to their sporadic counterpart, early identification of such high-risk individuals and their families is important in order to commence timely screening and surveillance programs.

Lynch syndrome is caused by germline mutations in DNA mismatch repair (MMR) genes *MLH1*, *MSH2*, *MSH6* and *PMS2*. Also genomic rearrangements within the epithelial cell adhesion molecule gene *EPCAM* can lead to silencing of the closely linked *MSH2* gene in *EPCAM*-expressing tissues. CRCs in Lynch syndrome are characterized by an adenoma-carcinoma progression ratio of 1:1 (estimated adenoma-cancer transformation time 1–3 years), as compared to sporadic cases that have a ratio of 30:1 (estimated adenoma-cancer transformation time 8–17 years) [2]. If left untreated, the majority of polyps will become malignant as observed in approximately 70% of patients at age 70 and 80% of patients at 85 years. There is an increased incidence of metachronous and synchronous colon cancers with a second primary CRC developing in up to 30% after 10 years and 50% after 15 years [2]. Lynch syndrome predisposes to extracolonic malignancies involving the endometrium, stomach, ovaries, small bowel, hepatobiliary epithelium, uroepithelial epithelium and brain [1,2].

The aim of this manuscript is to review the history and genetics of Lynch syndrome and provide a discussion pertaining to the clinical and molecular diagnostics, universal tumor screening and changes in testing paradigms.

## 2. History of Lynch Syndrome

The history of Lynch syndrome dates back to 1895 when Aldred Scott Warthin worked as the Chairman of the Department of Pathology at the University of Michigan, School of Medicine in Ann Arbor. He astutely observed his German seamstress to be depressed over the thought that she too would eventually succumb to gastric, colonic, or uterine cancer similar to the other members of her family. Indeed, she did die of endometrial cancer at an early age. Warthin studied her family in detail and published this large pedigree of 10 affected family members in 1913 outlining many generations affected by colonic, gastric and uterine cancers [3,4]. He performed an audit of 3600 cancer cases diagnosed in his laboratory between 1895 and 1912 and observed that approximately 15% of those had a positive family history of carcinoma. Warthin concluded that there was “some influence of heredity on cancer” [5]. An updated report on Family G was published in 1925. There was a higher familial preponderance for cancers of the gastrointestinal tract and uterus. Such cancers affected family members at a median age of 37.9 years and had a tendency for CRC to develop in the proximal colon. Warthin died in 1931 [5].

In 1966, Henry Lynch described two families from Nebraska (N) and Michigan (M) that had similar cancer patterns involving multi-generations that were akin to the original Family G. He studied the data from over 650 Family G members and later published his “Cancer Family ‘G’ Revisited” manuscript in 1971 [6] that solidified the evidence which characterized this syndromic disease as having an autosomal dominant inheritance pattern and an early age of onset (average age at onset <45 years) and involving adenocarcinomas of the colon, endometrium, and stomach [5]. Several other similar reports describing HNPCC were published in the mid-1980s and a number of clinical classification schemas were developed for research purposes [7,8,9,10].

In 1989, the International Collaborative Group on HNPCC (ICG-HNPCC) was established which developed a set of criteria known as the “Amsterdam criteria-I” for the diagnosis of HNPCC to facilitate identification of causative genes [11]. This was further broadened in 1999 to incorporate extracolonic tumours and was known as “Amsterdam criteria-II” [12]. With the identification of several mutations within the MMR genes (*MLH1*, *MSH2*, *MSH6*, and *PMS2*), the National Cancer Institute held an International Workshop on Lynch syndrome in Bethesda in November, 1997 [13]. They reported a standardized diagnostic panel of microsatellite markers and developed the Bethesda Guidelines for selecting patients’ CRC for MSI analysis [13,14]. These guidelines were revised and published in 2004 to include family history and specific pathologic features of CRC such as signet ring cell features, Crohn’s like reaction, mucinous features and location of the tumor in the right colon [15] (Table 1). These general guidelines have limitations as many Lynch syndrome families will not meet the Amsterdam Criteria or the Bethesda Guidelines. Conversely, despite meeting such criteria or guidelines, some families will not possess germline alterations in any DNA MMR genes. In 2008, Hampel *et al.* demonstrated the feasibility of large-scale immunohistochemistry (IHC) that could aid in directing genetic testing [1]. In 2009, the Jerusalem Workshop recommended routine MSI testing or immunohistochemistry for all CRCs diagnosed in patients below the age of 70 years [16]. These recommendations were incorporated into the Evaluation of Genomic Application in Practice and Prevention (EGAPP) evidence report [17].

**Table 1:** Breakdown of the Amsterdam Criteria I+II and Revised Bethesda Guidelines [11,12,13,14,15].

Amsterdam Criteria I At least three relatives with histologically verified colorectal cancer: One is a first-degree relative of the other two; At least two successive generations affected; At least one of the relatives with colorectal cancer diagnosed at <50 years of age; Familial adenomatous polyposis (FAP) has been excluded.
Amsterdam Criteria II At least three relatives with an hereditary nonpolyposis colorectal cancer (HNPCC)-associated cancer [colorectal cancer, endometrial, stomach, ovary, ureter/renal pelvis, brain, small bowel, hepatobiliary tract, and skin (sebaceous tumours)]: One is a first-degree relative of the other two; At least two successive generations affected; At least one of the syndrome-associated cancers should be diagnosed at <50 years of age; FAP should be excluded in any colorectal cancer cases; Tumors should be verified whenever possible.
Revised Bethesda Guidelines Colorectal tumors from individuals should be tested for MSI in the following situations: Colorectal cancer diagnosed in a patient who is <50 years of age. Presence of synchronous or metachronous colorectal, or other HNPCC-associated tumors regardless of age. Colorectal cancer with microsatellite instability-high (MSI-H) histology diagnosed in a patient who is <60 years of age. Colorectal cancer diagnosed in one or more first-degree relatives with an HNPCC-related tumor, with one of the cancers being diagnosed under age 50 years. Colorectal cancer diagnosed in two or more first- or second-degree relatives with HNPCC-related tumors, regardless of age.

## 3. Genetics of Lynch Syndrome

The majority of CRC develop sporadically from somatic alterations in colon epithelial cells; however, in up to 30% of cases, CRC develops in patients that have a strong family history. Patients with affected first-degree relatives have a 2–10 times increased risk of developing CRC and in the absence of a Lynch or polyposis syndrome probably harbor incompletely penetrant variants in a range of genes. Lynch syndrome patients have inherited at least one defective allele of a MMR gene. The normal function of the MMR proteins is to proofread the nucleotide sequence for potential base-base errors that occur during DNA synthesis. Microsatellites are short repetitive sequences that are distributed throughout the human genome. Defective MMR causes variations within the microsatellites, manifesting as a gain or loss in repeat length. This is described as microsatellite instability (MSI) [18,19,20,21]. Cancers that possess more than 40% microsatellite variations (*i.e.*, positive for two or more of the five standard microsatellite markers routinely tested) are described as high frequency MSI (MSI-H). Interestingly, this phenotype is also observed in 15% of sporadic CRCs due to somatic methylation of the *MLH1* promoter region. Further genotyping for the *BRAF* somatic V600E mutation can be performed to confirm somatic occurrences of MSI. Mutations of the *BRAF* gene with methylation of *MLH1* are typical of sporadic CRC and are almost never seen in Lynch syndrome [22,23,24]. Tumors that have no MSI are microsatellite stable (MSS) and those that possess less than 40% microsatellite variations (*i.e.*, one out of the five standard markers showing microsatellite instability) are low frequency MSI (MSI-L), although the relevance of this group is uncertain and these tumours are not considered microsatellite unstable [22,23,24].

The majority of individuals with Lynch syndrome possess at least one pathogenic germline mutation of the MMR genes *MLH1*, *MSH2*, *MSH6* or *PMS2*. *MLH1* and *MSH2* genes are by far the most commonly mutated in Lynch syndrome patients accounting for ~70% of the mutations identified (32% in *MLH1* and 38% in *MSH2*) [25,26]. Individuals who carry mutations in the *MSH2* gene have a preponderance for developing extracolonic cancer and a lower frequency of CRC when compared with *MLH1* [27,28]. *MSH6* mutations are commonly linked with gastrointestinal and endometrial cancer, and a later age of presentation [29,30]. *MSH6* is also recognized as a frequent cause of atypical Lynch syndrome (*i.e.*, not fulfilling the Amsterdam criteria) [29,30]. Senter *et al.* analysed 99 probands diagnosed with Lynch syndrome associated tumors showing isolated loss of PMS2 and demonstrated germline PMS2 mutation in 62% of probands [31]. Among families with monoallelic PMS2 mutations, 65.5% met revised Bethesda guidelines and the penetrance for monoallelic mutation carriers was lower than for the other MMR genes [31].

Recently, constitutional 3' deletions of *EPCAM* have been shown to cause Lynch syndrome through epigenetic silencing of *MSH2* in *EPCAM*-expressing tissues, resulting in tissue-specific *MSH2* deficiency [32]. Kempers *et al.* performed a cohort study comparing 194 patients carrying the *EPCAM* deletion to 473 patients carrying a mutation in *MLH1*, *MSH2*, *MSH6*, or a combined *EPCAM-MSH2* deletion. Carriers of an *EPCAM* deletion had a 75% cumulative risk of colorectal cancer before the age of 70 years, which did not differ significantly from that of carriers of combined *EPCAM-MSH2* deletion or mutations in *MSH2*, but was higher than noted for carriers of *MSH6* mutation. Only those with deletions extending close to the *MSH2* promoter had an increased risk of endometrial cancer. Therefore, these results underscore the effect of mosaic *MSH2* deficiency, leading to variable cancer risks, and could form the basis of an optimized protocol for the recognition and targeted prevention of cancer in *EPCAM* deletion carriers [33].

So far, genome wide association studies have identified approximately 20 gene variants associated with the development of sporadic CRC [34]. Wijnen *et al.* have identified the single nucleotide polymorphism rs16892766 (8q23.3) and rs3802842 (11q23.1) to be significantly associated with CRC risk in Lynch syndrome families [35]. For rs16892766, possession of the C-allele was associated with an elevated risk of CRC in a dose-dependent fashion, with homozygosity for CC being associated with a 2.16-fold increased risk. For rs3802842, the increased risk of CRC associated with the C-allele was only found among female carriers, while CRC risk was substantially higher among homozygous compared to the heterozygous carriers of the C-allele. In an additive model of both variants, the risk was significantly associated with the number of risk alleles. The effects were stronger in female carriers than in male carriers. Such modifiers may aid in identifying high-risk individuals who require more intensive surveillance [35].

Interestingly, of all the families who meet the Amsterdam-1 criteria, approximately 80% of families carry a hereditary abnormality in a MMR gene. In order to determine whether cancer risk in Amsterdam-1 families with no apparent deficiency in DNA MMR was different from cancer risk in Amsterdam-1 families with DNA MMR abnormalities, Lindor *et al.* identified 161 Amsterdam-1 pedigrees and compared families with and without MMR abnormalities [36]. Families who fulfilled the Amsterdam criteria-1 and did not possess a DNA MMR defect did not share the same cancer incidence as families with hereditary MMR deficiency. Relatives in such families carried a modest risk of developing CRC at an older age without extracolonic malignancies when compared to those who have an identifiable mismatch repair gene defects. Therefore, in order to distinguish these cohorts apart, the designation of “familial CRC type X” was suggested for this type of familial aggregation of CRC [36].

The Human Variome Project has recently established a pilot project in conjunction with the International Society for Gastrointestinal Hereditary Tumours (InSiGHT) in order to interrogate all inherited variation affecting colon cancer susceptibility genes. Genotypic-phenotypic data are stored in the InSiGHT Colon Cancer Gene Variant Databases [37]. This registry provides a deeper understanding into both rare and common forms of hereditary CRC syndromes [38]. Recently, InSiGHT formed an international panel of researchers and clinicians to review MMR gene variants submitted to the database in an effort to develop, test and apply a five-tiered scheme to classify 2360 unique constitutional MMR gene variants [39]. Out of the 12,006 variant entries in the InSiGHT database, the final outcome of standardized five-tiered InSiGHT classification of constitutional MMR gene variants included 2641 variants as Class 5 (pathogenic), 239 as Class 4 (probably pathogenic), 6982 as Class 3 (unknown), 53 as Class 2 (probably no pathogenicity) and 2091 variants as Class 1 (no known pathogenicity). This is the first large-scale comprehensive classification effort undertaken for the curation of a locus-specific database (LSDB) and providing summary information to assign variant pathogenicity. The InSiGHT initiative has provided a key model of LSDB-centric multidisciplinary collaboration for transparent interpretation of DNA variants. The criteria developed provide the basis for standardizing clinical classification of variants that will aid to inform patient and family management through genetic counseling [37,39].

## 4. Screening for Lynch Syndrome

Hereditary colorectal cancer syndromes encompass a spectrum of diseases that are caused by an equally diverse number of germline mutations. Although Lynch syndrome accounts for up to 5% of CRCs, affected individuals carry up to 80% lifetime risk for developing CRC and other cancers at an average age of 46 years [1,2]. This has significant public health implications especially in regards to patient selection for timely screening.

Several guidelines pertaining to familial CRC surveillance and screening have been proposed by a number of bodies [40,41,42,43], the largest of which being the National Comprehensive Cancer Network guidelines (NCCN) [43]. Germline MMR gene mutations are only detected in up to 80% of Lynch syndrome instances despite meeting the Amsterdam criteria and showing high MSI/loss of MMR protein expression [44,45,46,47]. The syndrome is now an amalgam of a number of molecular entities and even with the introduction of the revised Bethesda guidelines, up to 28% of MMR gene mutation carriers are missed [15,48]. The Amsterdam and Bethesda guidelines have been criticized for lacking in specificity and sensitivity thereby making them difficult to implement in clinical practice [34]. Other studies have also shown that the Bethesda guidelines may miss 6%–25% of mutation carriers [49,50]. Therefore, clinicians should make a major effort to document detailed family histories in order to identify families with Lynch syndrome.

Much debate exists regarding testing for MSI status by PCR only or MMR protein expression by IHC as there is a strong concordance between the two. Immunohistochemical staining provides a rapid, cost effective way for assessing the protein expression of the *MLH1*, *MSH2*, *MSH6*, and *PMS2* genes. Should one or more of these MMR proteins not stain, germline genetic testing can then be targeted to that particular gene [51,52]. To assess Lynch syndrome tumor screening programs and identify barriers to implementation, an electronic survey of the National Society of Genetic Counselors Cancer Special Interest Group was conducted in July 2011 [44]. Routine Lynch syndrome tumor screening protocols for newly diagnosed colon and/or endometrial cancers were set in place according to 52.8% of respondents and approximately half of these utilized a universal approach. Much variation existed regarding tumor screening methods; 64.2% started with immunohistochemistry, 20.8% started with MSI testing and 15.1% performed both on newly diagnosed CRC. Only 21.7% of respondents had a tumor-screening program in place for newly diagnosed endometrial cancers. Written consent was rarely obtained (7.1%) and there was a lack of uniformity in the way patients received their results. Several barriers to implementation were concerns regarding cost, bringing key players together and convincing medical staff of the necessity [44].

Overall, Lynch syndrome tumor screening programs are available however protocols vary widely. The current algorithm of making the diagnosis of Lynch syndrome involves meeting the criteria laid out in the Bethesda guidelines. If such criteria are not met, some authors feel there is little to no advantage in performing further genetic tests [22]. Conversely others advocate the routine screening of CRC pathology specimens for MSI and immunohistochemical expression of MMR [53,54]. This approach has been shown to have a better rate of identifying mutation carriers as compared to relying solely on the Bethesda guidelines. The Evaluation of Genomic Applications in Practice and Prevention (EGAPP) Working Group have advocated Lynch syndrome screening on all newly diagnosed CRC patients for the benefit of identifying at-risk relatives [17]. Recently, the revised guidelines for the clinical management of Lynch syndrome recommended that all CRC (<70 years) and all endometrial cancers (<70 years) should be tested by immunohistochemistry or MSI for the identification of patients potentially with Lynch syndrome [34]. These recommendations have been incorporated in the updated NCCN guidelines for Genetic/Familial-Risk Assessment: Colorectal Version I.2014 [43].

## 5. Conclusions

Despite the imperfections of current genetic testing, clinical judgment should dictate management plans and at risk patients should be enrolled in regular CRC surveillance programs. Patients who have a confirmed MMR gene mutation should undergo colonoscopy every 1–2 years beginning at the age of 20–25, or 10 years before the earliest age of onset in the family [44]. There has been a steady increase in Lynch syndrome tumor screening programs since 2000 and institutions are rapidly adopting a universal screening approach. It is time to standardize institutional high-risk/hereditary CRC clinics. International guidelines should determine the requirements and quality standards for establishing these clinics.
